# Supplementary figures and images for: Association between HLA-C alleles and COVID-19 severity in a pilot study with a Spanish Mediterranean Caucasian cohort
Source: PLoS One. 2022 Aug 12;17(8):e0272867. doi: 10.1371/journal.pone.0272867 (PMC9374209; doi:10.1371/journal.pone.0272867)

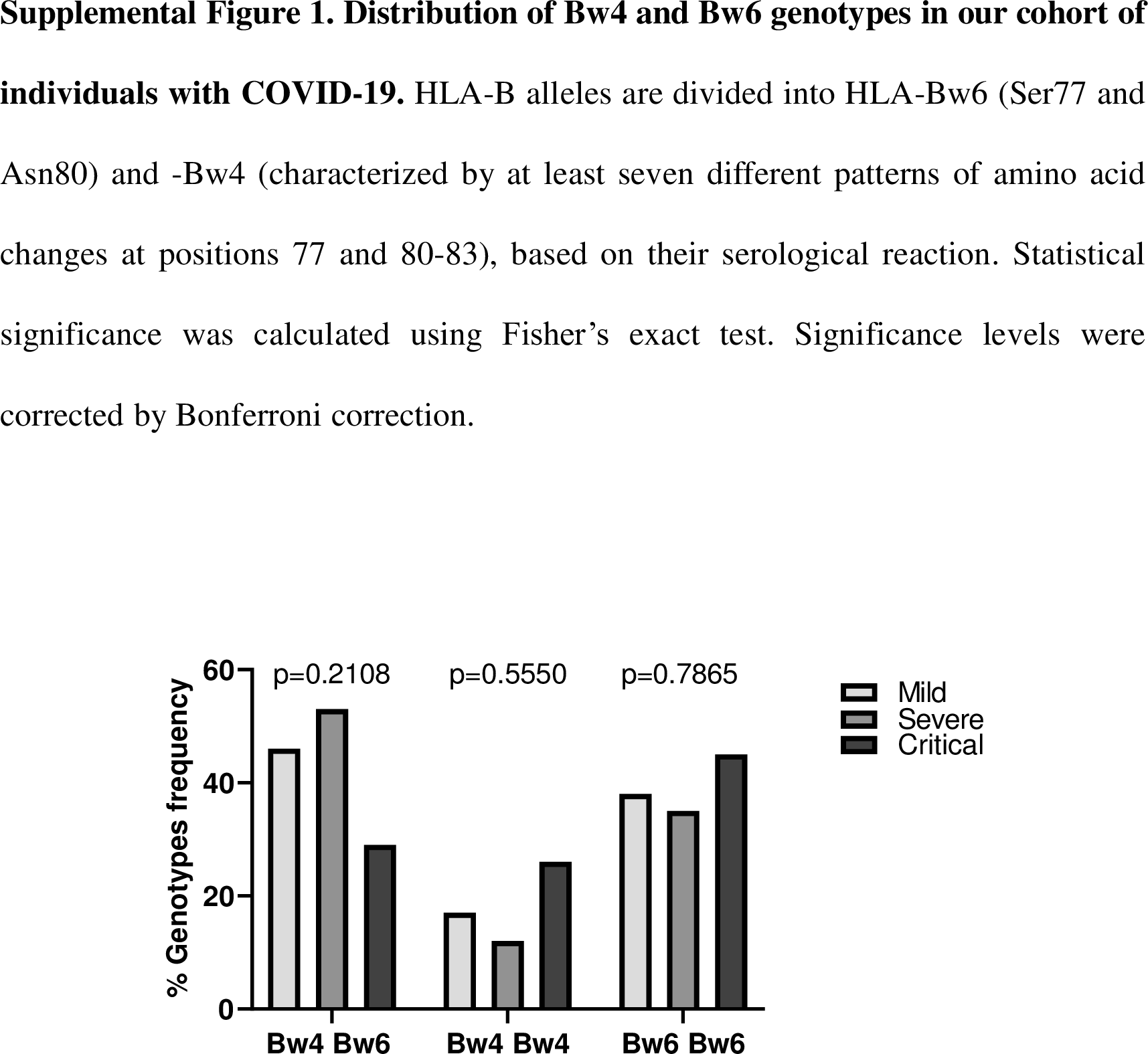

Supplement: S1 Fig — HLA-B alleles are divided into HLA-Bw6 (Ser77 and Asn80) and -Bw4 (characterized by at least seven different patterns of amino acid changes at positions 77 and 80–83), based on their serological reaction. Statistical significance was calculated using Fisher’s exact test. Significance levels were corrected by Bonferroni correction. (TIF) [file pone.0272867.s001.tif]

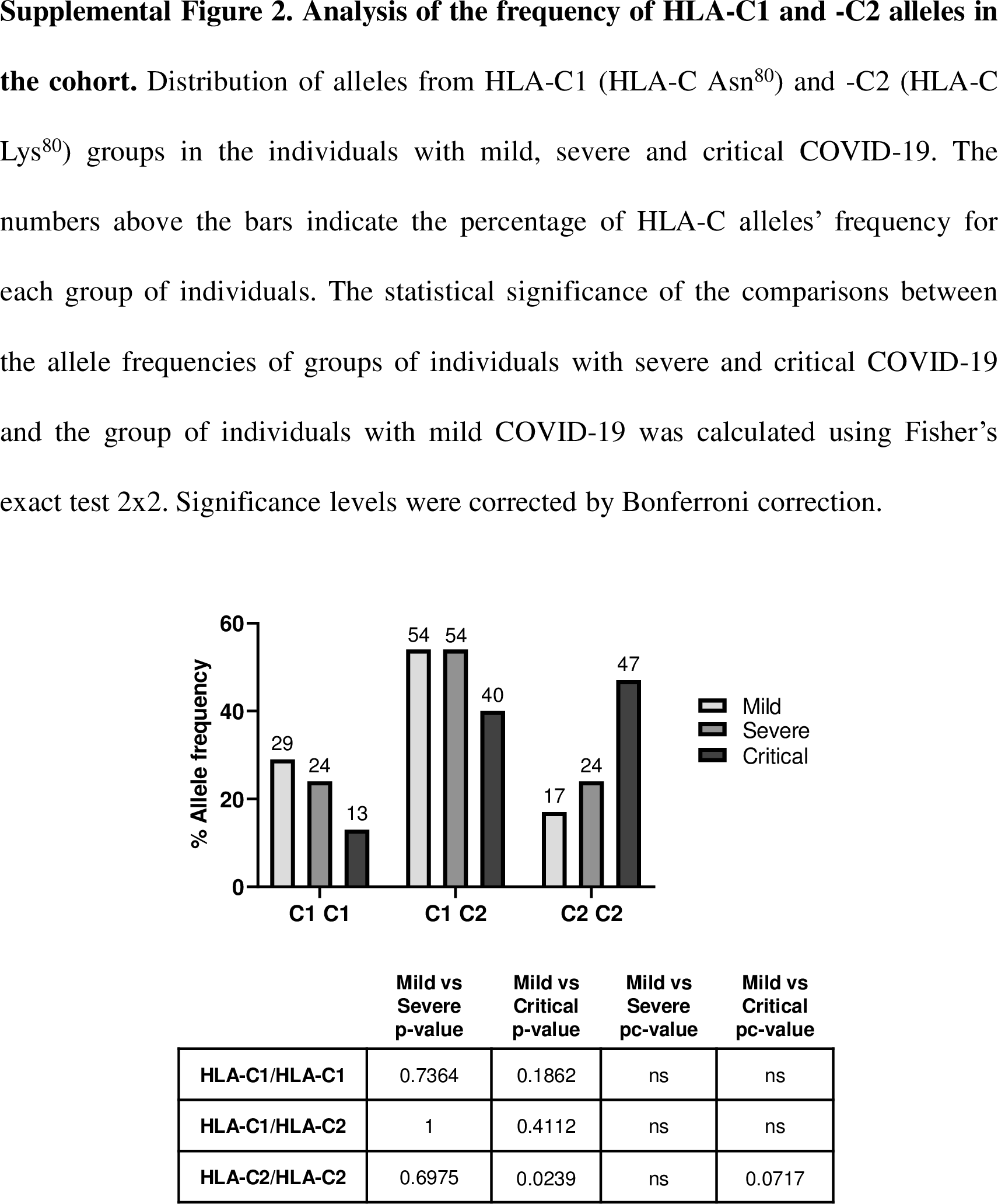

Supplement: S2 Fig — Distribution of alleles from HLA-C1 (HLA-C Asn80) and -C2 (HLA-C Lys80) groups in the individuals with mild, severe and critical COVID-19. The numbers above the bars indicate the percentage of HLA-C alleles’ frequency for each group of individuals. The statistical significance of the comparisons between the allele frequencies of groups of individuals with severe and critical COVID-19 and the group of individuals with mild COVID-19 was calculated using Fisher’s exact test 2x2. Significance levels were corrected by Bonferroni correction. (TIF) [file pone.0272867.s002.tif]

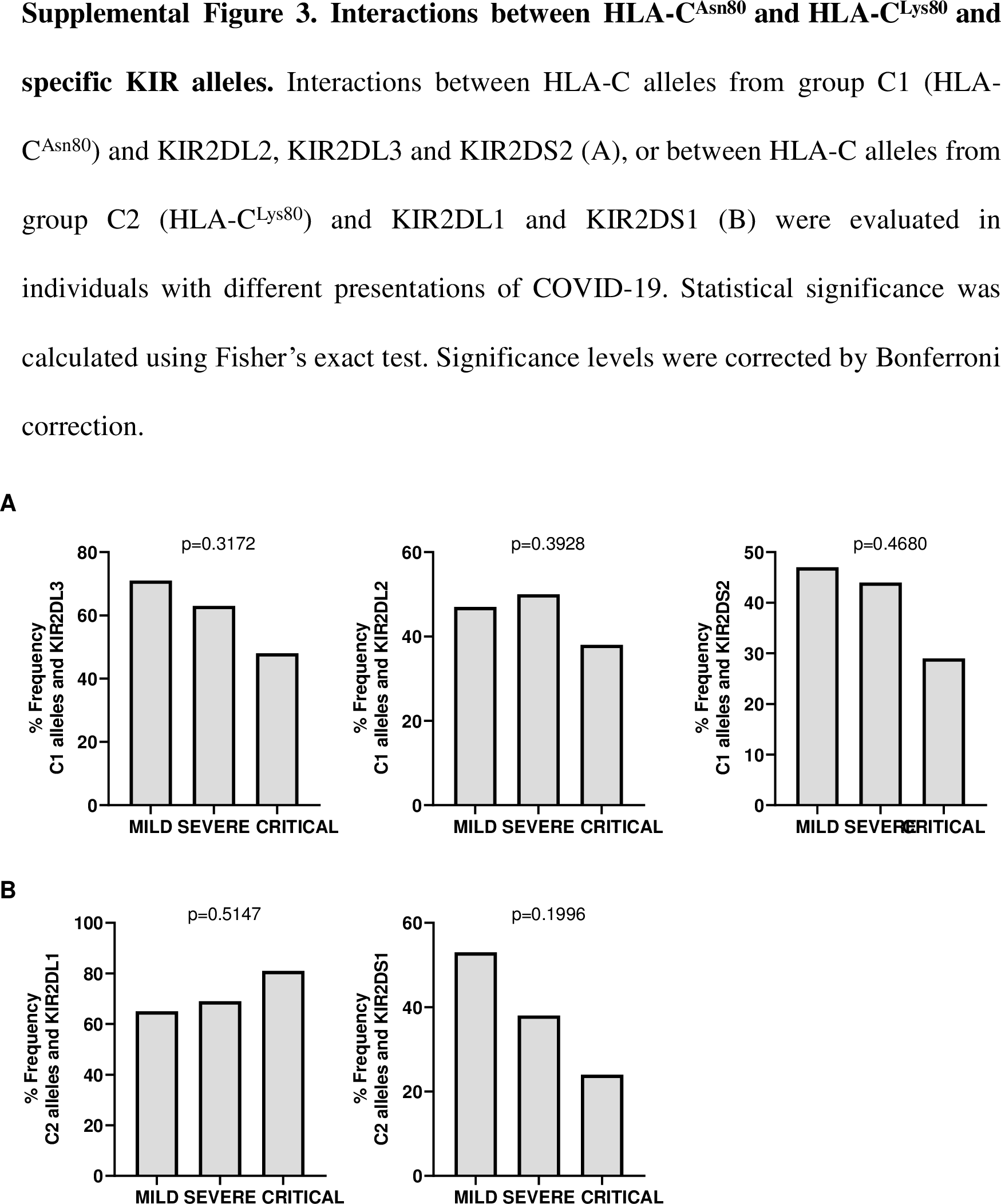

Supplement: S3 Fig — Interactions between HLA-C alleles from group C1 (HLA-CAsn80) and KIR2DL2, KIR2DL3 and KIR2DS2 (A), or between HLA-C alleles from group C2 (HLA-CLys80) and KIR2DL1 and KIR2DS1 (B) were evaluated in individuals with different presentations of COVID-19. Statistical significance was calculated using Fisher’s exact test. Significance levels were corrected by Bonferroni correction. (TIF) [file pone.0272867.s003.tif]
