# Supplementary material for: Association between HLA-C alleles and COVID-19 severity in a pilot study with a Spanish Mediterranean Caucasian cohort
Source: PLoS One. 2022 Aug 12;17(8):e0272867. doi: 10.1371/journal.pone.0272867 (PMC9374209; doi:10.1371/journal.pone.0272867)
Supplement: S1 Table — (DOCX) [file pone.0272867.s004.docx]

**Supplemental Table 1.** Demographic and clinical characteristics of homebound patients with mild COVID-19 who were recruited for this study at the Primary Healthcare Center Laín Entralgo (Alcorcón, Madrid, Spain).

| **ID** | **Days from clinical onset to sample** | **Exitus** | **Cough or Expectoration** | **Dyspnoea** | **Fever** | **Pneumonia** | **Diarrhea & Vomiting** | **Lethargy** | **Migraine** | **Asthenia** | **Treatment** | **LMWH** | **DM** | **DL** | **HTA** |
| --- | --- | --- | --- | --- | --- | --- | --- | --- | --- | --- | --- | --- | --- | --- | --- |
| 1 | 75 | NO | YES | YES | UD | NO | UD | NO | NO | YES | HCQ | NO | YES | YES | NO |
| 2 | 79 | NO | YES | NO | YES | NO | NO | NO | NO | YES | N/A | NO | NO | NO | NO |
| 3 | 90 | NO | NO | YES | UD | NO | YES | NO | NO | YES | N/A | NO | NO | NO | NO |
| 4 | 82 | NO | YES | YES | YES | NO | NO | NO | NO | YES | HCQ | NO | NO | NO | NO |
| 5 | 66 | NO | NO | NO | YES | NO | YES | UD | YES | YES | N/A | NO | NO | YES | NO |
| 6 | 83 | NO | YES | YES | NO | NO | NO | NO | NO | YES | N/A | NO | NO | NO | NO |
| 7 | UD | NO | YES | NO | NO | NO | YES | YES | NO | YES | N/A | NO | NO | NO | NO |
| 8 | 80 | NO | YES | NO | YES | NO | NO | NO | NO | NO | HCQ | NO | NO | YES | NO |
| 9 | UD | NO | NO | NO | UD | NO | NO | NO | NO | NO | N/A | NO | NO | NO | YES |
| 10 | 107 | NO | NO | NO | UD | NO | NO | NO | NO | NO | N/A | NO | NO | NO | NO |
| 11 | UD | NO | NO | NO | UD | NO | NO | NO | NO | NO | N/A | NO | NO | NO | NO |
| 12 | 95 | NO | NO | YES | YES | NO | YES | NO | NO | YES | HCQ | NO | NO | YES | NO |
| 13 | 95 | NO | YES | NO | YES | NO | YES | NO | NO | YES | N/A | NO | NO | YES | YES |
| 14 | 95 | NO | YES | NO | YES | NO | NO | NO | NO | YES | N/A | NO | NO | YES | NO |
| 15 | 88 | NO | YES | YES | YES | NO | YES | NO | YES | YES | N/A | NO | NO | NO | NO |
| 16 | 88 | NO | YES | NO | YES | NO | NO | NO | YES | YES | N/A | NO | NO | NO | NO |
| 17 | 94 | NO | YES | YES | YES | NO | YES | YES | YES | YES | N/A | NO | NO | NO | NO |
| 18 | UD | NO | NO | NO | YES | NO | NO | NO | NO | NO | N/A | NO | NO | NO | NO |
| 19 | 97 | NO | NO | NO | YES | NO | NO | NO | YES | YES | N/A | NO | NO | NO | NO |
| 20 | 36 | NO | YES | NO | YES | NO | YES | NO | NO | YES | N/A | NO | NO | NO | NO |
| 21 | UD | NO | YES | YES | YES | YES | NO | NO | YES | YES | N/A | NO | NO | NO | NO |
| 22 | 89 | NO | YES | NO | NO | NO | YES | NO | YES | YES | N/A | NO | NO | NO | NO |
| 23 | 95 | NO | NO | NO | NO | NO | YES | NO | YES | YES | N/A | NO | NO | NO | YES |
| 24 | UD | NO | NO | NO | UD | NO | NO | NO | NO | NO | N/A | NO | NO | NO | YES |

DL: dyslipidemia; DM: Diabetes mellitus, F: female; HCQ, Hydroxychloroquine; HTA: hypertension; LMWH: Low-molecular-weight heparin; M: male; N/A: Not applicable; UD: Undetermined.
