# Supplementary material for: Association between HLA-C alleles and COVID-19 severity in a pilot study with a Spanish Mediterranean Caucasian cohort
Source: PLoS One. 2022 Aug 12;17(8):e0272867. doi: 10.1371/journal.pone.0272867 (PMC9374209; doi:10.1371/journal.pone.0272867)
Supplement: S2 Table — (DOCX) [file pone.0272867.s005.docx]

**Supplemental Table 2.** Demographic and clinical characteristics of hospitalized patients with severe and critical COVID-19 who were recruited for this study at the Hospital Universitario Ramon y Cajal, Hospital Universitario Puerta de Hierro and Hospital de El Escorial (Madrid, Spain).

| **ID** | **COVID-19**  **presentation** | **Days from clinical onset to sample** | **Exitus** | **Cough or expectoration** | **Dyspnea** | **Fever** | **Pneumonia** | **Diarrhea and vomiting** | **Lethargy** | **Migraine** | **Asthenia** | **Treatment** | **LMWH** | **DM** | **DL** | **HTA** | **DIC** |
| --- | --- | --- | --- | --- | --- | --- | --- | --- | --- | --- | --- | --- | --- | --- | --- | --- | --- |
| 25 | Severe | UD | NO | UD | UD | YES | YES | UD | UD | UD | UD | HCQ, LPV/r, AZM | NO | NO | NO | NO | NO |
| 26 | Severe | 28 | NO | YES | NO | YES | NO | NO | NO | NO | YES | HCQ, AZM | NO | NO | NO | YES | NO |
| 27 | Severe | 40 | NO | YES | NO | YES | YES | NO | NO | NO | NO | HCQ , LPV/r | NO | YES | YES | YES | YES |
| 28 | Severe | 10 | NO | NO | NO | YES | YES | NO | NO | NO | NO | HCQ , LPV/r, AZM | YES | NO | YES | NO | YES |
| 29 | Severe | 18 | NO | NO | NO | YES | YES | NO | NO | NO | YES | HCQ, LPV/r, AZM | YES | NO | NO | NO | NO |
| 30 | Severe | UD | NO | UD | YES | UD | YES | UD | YES | UD | UD | HCQ, AZM | YES | YES | NO | YES | YES |
| 31 | Severe | 32 | NO | YES | YES | YES | NO | NO | NO | NO | NO | HCQ, AZM | NO | YES | YES | NO | NO |
| 32 | Severe | 1 | NO | YES | YES | NO | YES | YES | NO | NO | NO | HCQ, AZM | NO | NO | NO | NO | NO |
| 33 | Severe | 42 | NO | YES | NO | NO | YES | NO | NO | NO | NO | LPV/r, AZM | NO | NO | NO | YES | NO |
| 34 | Severe | 52 | NO | YES | NO | NO | YES | NO | NO | YES | NO | HCQ, LPV/r, AZM | NO | NO | NO | YES | NO |
| 35 | Severe | 37 | NO | YES | NO | NO | YES | NO | NO | NO | NO | HCQ, AZM | YES | YES | NO | YES | NO |
| 36 | Severe | UD | NO | NO | NO | NO | YES | NO | NO | NO | NO | HCQ, AZM | NO | NO | YES | YES | NO |
| 37 | Severe | 17 | NO | NO | NO | YES | YES | NO | NO | NO | NO | HCQ | NO | NO | NO | YES | YES |
| 38 | Severe | UD | NO | NO | NO | NO | YES | YES | NO | NO | NO | HCQ | UD | NO | NO | YES | YES |
| 39 | Severe | 3 | NO | YES | NO | NO | YES | NO | NO | NO | NO | HCQ | NO | NO | NO | YES | NO |
| 40 | Severe | UD | NO | 90 | YES | NO | NO | NO | NO | NO | NO | HCQ, AZM | YES | NO | NO | NO | NO |
| 41 | Severe | 9 | NO | YES | YES | YES | YES | YES | YES | YES | YES | MER, LZD, DXM | NO | NO | YES | YES | NO |
| 42 | Critical | 1 | YES | UD | UD | UD | UD | UD | UD | UD | UD | UD | UD | UD | UD | UD | NO |
| 43 | Critical | 62 | NO | YES | YES | YES | YES | NO | NO | YES | NO | HCQ , LPV/r, AZM | YES | NO | YES | YES | NO |
| 44 | Critical | 55 | NO | NO | YES | YES | NO | NO | NO | NO | NO | HCQ , LPV/r, AZM | YES | NO | NO | YES | NO |
| 45 | Critical | 38 | NO | YES | YES | YES | YES | YES | NO | YES | YES | HCQ , LPV/r, AZM | YES | NO | NO | NO | NO |
| 46 | Critical | 18 | NO | YES | NO | NO | YES | NO | NO | NO | NO | HCQ , LPV/r | YES | NO | NO | NO | NO |
| 47 | Critical | 55 | NO | YES | YES | YES | YES | NO | NO | YES | NO | HCQ , LPV/r, AZM | YES | NO | NO | NO | NO |
| 48 | Critical | 50 | YES | YES | YES | YES | YES | NO | NO | YES | NO | HCQ , LPV/r, AZM | YES | NO | YES | NO | NO |
| 49 | Critical | 53 | YES | YES | YES | YES | YES | NO | NO | NO | NO | HCQ , LPV/r, AZM | YES | YES | YES | NO | NO |
| 50 | Critical | UD | YES | YES | NO | NO | YES | NO | NO | YES | NO | HCQ , LPV/r, AZM | YES | NO | NO | NO | NO |
| 51 | Critical | 46 | YES | NO | NO | NO | NO | NO | NO | NO | NO | HCQ | YES | NO | NO | NO | NO |
| 52 | Critical | 34 | YES | YES | YES | YES | YES | NO | NO | YES | NO | LPV/r, AZM | YES | NO | NO | YES | NO |
| 53 | Critical | 53 | YES | YES | YES | YES | YES | NO | NO | NO | NO | HCQ , LPV/r, AZM | YES | NO | NO | YES | NO |
| 54 | Critical | UD | NO | UD | UD | UD | UD | UD | UD | UD | UD | UD | YES | NO | NO | NO | UD |
| 55 | Critical | 14 | NO | YES | YES | YES | YES | NO | NO | YES | NO | HCQ , LPV/r, AZM | YES | NO | NO | NO | NO |
| 56 | Critical | 30 | YES | NO | YES | YES | NO | NO | NO | NO | YES | HCQ | YES | NO | NO | YES | YES |
| 57 | Critical | 59 | NO | YES | YES | YES | YES | NO | NO | YES | NO | HCQ , LPV/r, AZM | YES | NO | YES | YES | NO |
| 58 | Critical | 19 | NO | YES | YES | YES | YES | NO | NO | NO | NO | HCQ , LPV/r, AZM | YES | NO | YES | NO | NO |
| 59 | Critical | UD | NO | NO | NO | NO | YES | NO | NO | NO | NO | HCQ , LPV/r, AZM | YES | NO | NO | YES | NO |
| 60 | Critical | 71 | NO | UD | UD | YES | YES | YES | NO | NO | NO | HCQ, AZM | NO | UD | UD | UD | NO |
| 61 | Critical | UD | YES | NO | NO | NO | YES | NO | NO | NO | YES | HCQ, AZM | NO | NO | NO | YES | NO |
| 62 | Critical | 11 | NO | YES | YES | YES | YES | NO | YES | NO | YES | NO | YES | NO | YES | NO | NO |
| 63 | Critical | 42 | YES | YES | YES | YES | YES | YES | NO | NO | YES | NO | YES | NO | YES | NO | NO |
| 64 | Critical | 28 | YES | YES | YES | YES | YES | NO | YES | NO | NO | MER, PIR | NO | NO | YES | NO | NO |
| 65 | Critical | 30 | NO | NO | NO | YES | YES | YES | NO | NO | NO | PIR | YES | NO | YES | YES | NO |
| 66 | Critical | 35 | YES | YES | YES | YES | YES | NO | YES | NO | NO | VCZ, MER | NO | NO | YES | YES | NO |
| 67 | Critical | 15 | NO | YES | YES | YES | YES | NO | NO | NO | YES | PIP/TZ | NO | NO | NO | NO | NO |
| 68 | Critical | 34 | YES | YES | YES | YES | YES | NO | NO | NO | NO | MER | YES | NO | NO | NO | NO |
| 69 | Critical | 49 | NO | NO | NO | YES | YES | NO | NO | NO | NO | RDV | YES | NO | YES | YES | NO |
| 70 | Critical | 37 | NO | YES | YES | YES | YES | NO | NO | NO | NO | MER | YES | NO | YES | YES | NO |
| 71 | Critical | 28 | NO | YES | YES | YES | YES | NO | NO | NO | YES | NO | YES | NO | YES | YES | NO |
| 72 | Critical | 20 | YES | YES | YES | NO | YES | NO | NO | NO | NO | RDV, mPRED | NO | UD | YES | YES | NO |

AZM: azithromycin; CRO:ceftriaxone; CS: corticosteroids; DIC: Disseminated Intravascular Coagulation ; DL: Dyslipidemia; DM: Diabetes mellitus; DXM: Dexamethasone; F: Female HCQ: Hydroxychloroquine; HT: Hypertension; LMWH: Low-molecular-weight heparin; LPV/r: Lopinavir/ritonavir; LZD: Linezolid; M: Male; MER: Meropenem; mPRED: Methylprednisolone; PIP/TZ: Piperacillin/Tazobactam; PIR: Pirfenidone; RDV: Remdesevir;; TOZ: Tocilizumab; UD: Undetermined; VCZ: Voriconazol
